# Safeguarding Quality in Health and Medical Science Information Today

**DOI:** 10.1080/1179271X.2026.2655409

**Published:** 2026-04-10

**Authors:** Scott C. Ratzan, Rebecca K. Ivic, Kenneth H. Rabin, Ruth M. Parker, Sara Rubinelli, Rafael Obregon, Lawrence O. Gostin, Michael G. Baker, Samantha Thomas, Don Nutbeam, Ilona Kickbusch, J. Gregory Payne, Vivianne Ihekweazu, Kirsten J. McCaffery, Richard L. Street, Sir Cary Cooper

**Affiliations:** ^a^ Journal of Health Communication: International Perspectives; ^b^ JAMA; ^c^ AMWA Journal; ^d^ Health Promotion International; ^e^ Public Health Research & Practice; ^f^ American Behavioral Scientist; ^g^ Nigeria Health Watch; ^h^ Patient Education and Counseling; ^i^ Journal of Organizational Behavior; ^j^ Stress & Health

Health and medical journals have a mission to evaluate and disseminate information generated from rigorous and peer reviewed scientific enquiry. As journal editors, we are entrusted with safeguarding the integrity of the scientific record, supporting the researchers who advance discovery, and honoring the excellence of academic institutions that cultivate rigorous and unbiased inquiry. Maintaining the integrity of the information published in our journals helps ensure that scientists, health professionals, policymakers and the public can benefit from information that is accurate, trustworthy, accessible, and understandable.

As journal editors, we have a responsibility to provide and defend evidence-based information that helps shape public policy, inform clinical decisions, and guide people in making informed choices about their health,

Growing public and political skepticism of health information in several countries underscores the fragility of a foundation of trust in science that for decades had been widely accepted. For example, public health guidance from the US Centers for Disease Control and Prevention (CDC) was widely trusted for decades. That trust has now waned, if not collapsed [1]. Recent changes in governance as well as public guidance on subjects as diverse as immunization and the social determinants of health exemplify the impact of political interference. They also illustrate the public health consequences as the US (and other countries) experience increased outbreaks and fatalities from vaccine preventable disease.

When formerly authoritative public health agencies are politically influenced, public trust can erode, and health information becomes vulnerable to disappearance, distortion, and uncertainty. Once credible institutions can quickly be seen as unreliable sources of health information. The result is a public that is confused and often misled.

Across the history of ideas, moments of informational instability have carried deep civic and moral consequences. Thinkers from Plato and Aristotle to Saints Thomas Aquinas and Augustine warned that societies falter not only when institutions weaken, but when the distinction between episteme (knowledge grounded in method and evidence) and doxa (opinion shaped by sentiment or power) becomes blurred. Echoing Plato, individuals who mistake shadows for truths lose the capacity to judge reality.

Threats to the quality and integrity of health information today reflects these ancient concerns. History warns us that when the public can no longer rely on stable, verifiable sources, the moral bond of truth that holds communities together is jeopardized.

Our vulnerability has expanded in an era marked by the rapid rise of misinformation and Artificial Intelligence (AI)-generated falsehoods. The World Economic Forum’s *Global Risks Report* in 2025 and 2026 identified misinformation and disinformation as a leading global risk for the next two years, surpassing climate threats and financial instability [2,3]. Generative AI accelerates these risks by embedding low-quality or fabricated content in public search engines, further weakening the public’s ability to differentiate expert knowledge from distortions of it. But the challenge goes beyond AI. The entire global information ecosystem, fragmented, fast-moving, and increasingly unregulated, makes scientific integrity harder to preserve and maintain.

In the face of this troubling trend, we assert that quality health information must remain rooted in scientific integrity and must not deviate from the fundamental scientific methodology that produces, interprets, communicates, and applies it in practice. Several of the authors of this editorial are currently members of a new *Nature Medicine* Commission on Quality Health Information for All that defines quality health information as scientifically sound, accessible, clear, understandable, and essential for appropriate decision-making to improve health [4].

When we speak of quality health information, we refer not only to the scientific validity of its content but also to the ethical orientation that sustains it. The pursuit of quality is an act of care: care for truth, care for the public, care for the vulnerable, and care for a civil society who depend on trustworthy guidance to make informed decisions. We believe it is the thread that binds scientific rigor with civic duty and the common good.

Quality health information and communication are recognized as determinants of health-shaping clinical decisions, public behavior, equity, resilience, and trust. Importantly, it is the *quality of the information* that is communicated that determines whether it informs or misleads, empowers or confuses, strengthens or destabilizes, and ultimately helps shape the public’s health.

Scientific Integrity frameworks from WHO [5] and UNESCO [6] to COPE [7] and the ICMJE [8] offer reasonable levels of transparency, accountability, reproducibility, stability, and equitable access, which are structural requirements for safeguarding public health. The European Union [9,10,11] also is developing regulatory frameworks, including the Digital Services and Digital Markets Acts, which impose strict obligations on digital platforms to mitigate systemic risks, elevate authoritative sources, and strengthen traceability. The EU’s companion Artificial Intelligence Act introduces guardrails for transparency, data-quality, and requirements for human oversight.

Across these efforts runs a single, unifying principle: protecting the integrity of health information demands coordinated stewardship and sound governance. As editors with expertise across diverse scientific areas, we have seen that the expansion of digital content has allowed AI-generated scientific and health narratives to proliferate. We expect this trajectory to only intensify. If quality is to be maintained, we advocate for responsible oversight to harness the benefits of science and health.

It is important to emphasize that the same digital and AI technologies that often propagate false or misleading information also hold significant potential to strengthen information quality. When properly designed and governed, AI can enhance scientific integrity by detecting anomalies in manuscripts, identifying data fabrication, supporting peer review, tracking retractions, verifying citations, detecting plagiarism or image manipulation and enabling rapid synthesis of emerging evidence. AI-driven provenance tracking could alert clinicians and researchers when guidance changes, ensuring transparency and accountability for digital updates [12].

AI can help us identify misleading information, detect rumors, flag errors before they cause harm, and elevate robust evidence above the surrounding noise. It can increase the reach of high-quality research, strengthen peer-review systems, and protect vulnerable communities from harmful or incomplete information. AI can also help consumers of health information become more digital and health literate. Yet all of this is possible only with responsible stewardship, effective regulation, and vigilant oversight of digital platforms. Social media companies themselves owe a public duty to ensure the accuracy and reliability of their content.

AI offers editors further opportunities to improve quality across the research ecosystem. AI systems can help detect inconsistencies in manuscripts, flag statistical anomalies, identify duplicate or fabricated data, and scan a vast and growing literature more quickly than human reviewers. AI systems can also enhance public and professional resilience by identifying linguistic signatures of misinformation, flagging unsupported claims, and calling attention to potential conflicts of financial interest. AI could be employed as a tool for enhancing editorial rigor, strengthening peer review, and empowering clinicians and the public to navigate the information ecosystem with renewed confidence.

Yet the promise of AI will only be realized if global standards are established, recognized, and enforced. This requires coordination across multidisciplinary fields, sectors and across borders. Supporting scientific integrity and quality information is not solely the responsibility of journals or government agencies; it is a shared global obligation involving primary and secondary schools, universities, funders, researchers, publishers, professional societies, health systems, technology companies, and civil society.

Society must foster heightened digital literacy among the consumers of this information at all levels. Editorial work, at its best, is an expression of the capacity to judge complex evidence, to weigh uncertainty responsibly, and to communicate findings in ways that respect both scientific rigor and human vulnerability. It is also an expression of civic responsibility. The health of the state depends on the reliability of the knowledge circulating within it. In this vital context, health editors thus serve as custodians of this common good: protecting the continuity of knowledge, preserving archives from distortion, and ensuring that the public enjoys the benefits of verifiable reality.

The path forward means strengthening global standards, ensuring transparency in public communication, preserving archives, improving health literacy, protecting the independence of scientific institutions, and investing in the next generation of professionals who will produce, review, and disseminate evidence.

It is our editorial duty to advocate forcefully for all digital platforms and AI systems in the public sphere to be developed with safeguards that advance health and well-being that are rooted in sound science and global health norms. We also recognize our own responsibility to collaborate far more actively to protect the integrity of what we do.

Over the centuries, philosophers have insisted that truth is not self-sustaining. It requires the continued effort of individuals and institutions willing to uphold the fragile bond between evidence and meaning, knowledge and responsibility, science and the common good. The times demand that we reclaim that shared duty and act with a unity of purpose.

## References

[1] KFF. The Monitor: Tracking Health Information and Trust. KFF. 2026. https://www.kff.org/topic/health-information-trust/

[2] World Economic Forum. The Global Risks Report 2025, 20th Edition, Insight Report. 2025. https://reports.weforum.org/docs/WEF_Global_Risks_Report_2025.pdf

[3] World Economic Forum. The Global Risks Report 2026, 21st Edition, Insight Report. 2026. https://reports.weforum.org/docs/WEF_Global_Risks_Report_2026.pdf

[4] Ratzan SC, Larson HJ, Batista C, Kuzmanovic A, Kalb PE, Rabin KH, Gostin LO. The Quality Health Information for All Commission: Reinventing health communication for the digital era. Nat Med. 2025;31:22–23. doi:10.1038/s41591-024-03446-0 39843853

[5] World Health Organization. WHO policy on research integrity and responsible conduct of research. World Health Organization. 2024. https://www.who.int/about/ethics/code-of-conduct-for-responsible-research

[6] UNESCO. Recommendation on the ethics of artificial intelligence. United Nations Educational, Scientific and Cultural Organization. 2021. https://www.unesco.org/en/artificial-intelligence/ethics

[7] Committee on Publication Ethics (COPE). Guidance and tools from COPE (including AI and publication ethics). COPE. 2025. https://publicationethics.org/guidance

[8] International Committee of Medical Journal Editors (ICMJE). Recommendations for the Conduct, Reporting, Editing, and Publication of Scholarly Work in Medical Journals. 2025. https://www.icmje.org/recommendations/10.12771/emj.2024.e48 PMC12093628 40704003

[9] European Parliament and Council of the European Union. Regulation (EU) 2022/2065 on a single market for digital services (Digital Services Act). OJEU. 2022a. https://data.europa.eu/eli/reg/2022/2065/oj/eng

[10] European Parliament and Council of the European Union. Regulation (EU) 2022/1925 on contestable and fair markets in the digital sector (Digital Markets Act). OJEU. 2022b. https://eur-lex.europa.eu/eli/reg/2022/1925/oj

[11] European Parliament and Council of the European Union. Regulation (EU) 2024/1689 laying down harmonised rules on artificial intelligence (Artificial Intelligence Act). OJEU. 2024. https://artificialintelligenceact.eu/the-act/

[12] Pellegrina D, Helmy M. AI for scientific integrity: detecting ethical breaches, errors, and misconduct in manuscripts. Front Artif Intell. 2025;8:1644098. doi:10.3389/frai.2025.1644098 40964145 PMC12436494

